# Anti-Inflammatory Efficacy of Curcumin as an Adjunct to Non-Surgical Periodontal Treatment: A Systematic Review and Meta-Analysis

**DOI:** 10.3389/fphar.2022.808460

**Published:** 2022-01-24

**Authors:** Yang Zhang, Lei Huang, Jinmei Zhang, Alessandra Nara De Souza Rastelli, Jingmei Yang, Dongmei Deng

**Affiliations:** ^1^ Department of Periodical Press and National Clinical Research Center for Geriatrics, West China Hospital, Sichuan University, Chengdu, China; ^2^ Chinese Evidence-Based Medicine Center, West China Hospital, Sichuan University, Chengdu, China; ^3^ West China School of Public Health and West China Fourth Hospital, Sichuan University, Chengdu, China; ^4^ State Key Laboratory of Oral Disease and National Clinical Research Center for Oral Disease, Department of Periodontics, West China Hospital of Stomatology, Sichuan University, Chengdu, China; ^5^ Department of Restorative Dentistry, School of Dentistry, São Paulo State University-UNESP, Araraquara, Brazil; ^6^ Department of Preventive Dentistry, Academic Centre for Dentistry Amsterdam, University of Amsterdam and Vrije Universiteit Amsterdam, Amsterdam, Netherlands

**Keywords:** curcumin, anti-inflammatory, periodontal disease, non-surgical periodontal treatment (NPT), meta-analysis

## Abstract

**Objective:** Curcumin has been used as an adjunct to non-surgical periodontal treatment. However, the efficacy of curcumin in the periodontal therapy remained controversial. This study aimed to evaluate the anti-inflammatory efficacy of curcumin as an adjunct to non-surgical periodontal treatment (NPT) by systematic review.

**Methods:** Databases including Embase, PubMed, Cochrane Central Register of Controlled Trials (CENTRAL), and ClinicalTrials.gov were searched to identify relevant RCTs on the use of curcumin as an adjunct to NPT for the treatment of periodontal disease from inception to July 21, 2021. Two reviewers independently screened literature, extracted data and assessed the risk of bias of the included studies. Meta-analysis was then performed using Review Manager 5.3 software.

**Results:** A total of 18 RCTs involving 846 patients/sites were included in this meta-analysis. The results of the meta-analysis revealed that as compared to NPT alone, curcumin as an adjunct to NPT resulted in significant reduction in gingival index (GI) at the 1-week (mean differences (MD) = −0.15, 95% confidence intervals (CI) −0.26 to −0.05, *p* = 0.005), 2-week (MD = −0.51, 95%CI −0.74 to −0.28, *p* < 0.0001), 3-week (MD = −0.34, 95%CI −0.66 to −0.02, *p* = 0.03), 4-week (MD = −0.25, 95%CI −0.48 to −0.02, *p* = 0.04) or 6-week (MD = −0.33, 95%CI −0.58 to −0.08, *p* = 0.01) follow-ups. Similar significant reductions were also observed for sulcus bleeding index (SBI) at 1, 2, 4, and 12 weeks. However, there were no statistically significant differences in reducing bleeding on probing (BOP) between curcumin as an adjunct and NPT alone at 4, 12, and 24 weeks.

**Conclusion:** Based on the current evidence, curcumin demonstrates anti-inflammatory efficacies in terms of reducing GI and SBI compared with NPT alone. Moreover, curcumin is a natural herbal medicine with few side effects, and it is a good candidate as an adjunct treatment for periodontal disease.

## Introduction

Periodontal diseases, which include a range of conditions from gingivitis to periodontitis, are the most common chronic oral diseases affecting the majority of populations worldwide. This worldwide health problem has influenced 76% of the population in Europe and the US, ranking as the sixth most prevalent condition globally (Frencken et al., 2017). Dental plaque is the primary etiology attributed to this disease (Sanz et al., 2017), and the main goal of periodontal therapy is addressing the primary etiology. Traditionally, the main treatment modality for eliminating the infection is non-surgical periodontal therapy (NPT), including scaling for gingivitis and scaling and root planing (SRP) for periodontitis. NPT aims to reduce the periodontal pathogen invasion and manage the healing of periodontal tissue. However, the efficacy of NPT could be limited by several factors, such as deep periodontal pockets and complex root anatomy (Tomasi et al., 2007; Heitz-Mayfield and Lang, 2013). Therefore, antibiotics, such as amoxicillin, metronidazole, and tetracycline, have been introduced as adjuncts to mechanical debridement to enhance the efficacy of periodontal therapy (Petersilka et al., 2002; Slots and Ting, 2002). The application of antibiotics is debatable, since antimicrobial resistance has become a threat to global public health (Brinkac et al., 2017), the local application of antibiotics could even lead to oral bacterial resistance (Ahmadi et al., 2021).

Therefore, several alternative adjunctive drugs, especially natural agents, have been suggested as alternative antimicrobial methods. Curcumin, an age-old plant-derived polyphenol extracted from the rhizome of turmeric (Bisht et al., 2010), has become popular in the last 50 years due to its multiple therapeutic functions. Natural curcumin is defined as 1,7-bis-(4-hydroxy-3-methoxyphenyl)-hepta-1,6-diene-3,5-dione with a chemical formula of C_21_H_20_O_6_, according to the International Union of Pure and Applied Chemistry (IUPAC). Extensive research has shown that curcumin possesses anti-inflammatory, antioxidative, antiangiogenic, immunoregulatory, antibacterial, and proapoptotic properties (Pimentel et al., 2020), and curcumin has been proven to be effective in the treatment of rheumatoid arthritis (Conigliaro et al., 2019), inflammatory bowel disease (Sharma et al., 2019) and oral diseases (Tang et al., 2020), such as oral mucosal disease, oral lichen planus, oral squamous cell carcinoma and periodontal disease. Recently, a meta-analysis revealed that local delivery of curcumin showed similar clinical efficacies to chlorhexidine, the gold standard as an adjunct to SRP (Zhang et al., 2021).

However, whether curcumin could strengthen the effectiveness of NPT in periodontal therapy is still controversial. Some studies reported that curcumin, as an adjunctive treatment, could improve gingival inflammation (Gottumukkala et al., 2013; Guru et al., 2020; Mohammad, 2020), whereas other studies did not observe any improvement (Jalaluddin et al., 2019; Kaur et al., 2019; Pérez-Pacheco et al., 2021). Thus, this systematic review aims to perform a meta-analysis to explore whether curcumin as an adjunctive to NPT yields better clinical outcomes in terms of reducing periodontal inflammation than NPT alone.

## Materials and Methods

This systematic review was registered on the PROSPERO platform (registration number: CRD42021267612) and was conducted following the Preferred Reporting Items for Systematic Reviews and Meta-analyses (PRISMA) guidelines (Page et al., 2021).

### Search Strategy

We searched databases including Embase, PubMed, Cochrane Central Register of Controlled Trials (CENTRAL), and ClinicalTrials.gov without language restriction from inception to July 21, 2021, to identify relevant RCTs on the use of curcumin as an adjunct to NPT for the treatment of patients with periodontal disease. We combined MeSH and free text terms to identify the relevant articles. The search strategy is shown in the Supplementary Material. An additional search was performed among the references of the included studies to identify potentially eligible studies. We also manually searched the references of published reviews to collect additional relevant studies.

### Inclusion Criteria

Studies were included by applying the following population-intervention-comparator-outcomes-study design (PICOS): 1) Participants: Adult patients over 18 years of age diagnosed with periodontal disease. There were no restrictions on ethnicity or disease severity. 2) Interventions and comparisons: patients receiving curcumin (no restriction on dosage and form) as an adjunct to NRP in the intervention group and NPT alone as the control group. 3) Outcomes: The primary outcomes were gingival index (GI), sulcus bleeding index (SBI) and bleeding on probing (BOP). The secondary outcomes included plaque index (PI), microbiological indicators, inflammatory factors and adverse events. Studies reporting at least one primary outcome of interest with reliable and available data were included. 4) Study design: Randomized controlled trials (RCTs) were included in our study. There were no restrictions on the masking method or split-mouth design.

### Exclusion Criteria

The exclusion criteria were as follows: 1) Studies on systematic application of curcumin, not topical use in the oral cavity. 2) Studies included patients with systemic diseases. 3) Studies that included only patients who received other adjunct treatments, such as photodynamic therapy, other medications or surgical treatments. 4) Studies included patients who received periodontal treatment or antibiotic therapy prior to NPT. 5) *In vitro* or animal experiments. 6) Studies with incomplete data: targeted outcomes were not reported or could not be obtained after contacting authors. 7) Data were duplicated. 8) Trials were only reported as conference abstracts. 9) Studies were not reported in English.

### Data Extraction

Two reviewers independently screened titles, abstracts and full texts for eligible literature and then completed the data extraction. Disagreements were resolved by discussion or consultation with a third reviewer. The following data were extracted from each RCT: 1) Study characteristics: author name, year of publication, country of study, number of patients, and study design. 2) Patient characteristics: sex and age. 3) Interventions and comparisons: details of the curcumin treatment and NPT treatment groups (e.g., drug type, doses used, and duration of treatment). 4) Elements for the risk of bias assessment. 5) Outcomes: primary outcomes (GI, SBI and BOP) and secondary outcomes (PI, microbiological indicators, inflammatory factors and adverse events) at different follow-up time points. If a trial had multiple reports, the data from all sources were carefully examined for consistency.

### Risk of Bias Assessment

Two reviewers independently evaluated the risk of bias of the included RCTs according to the Cochrane Collaboration’s tool, which is described in the Cochrane Handbook (Higgins et al., 2020). Seven domains were assessed: 1) random sequence generation; 2) allocation concealment; 3) blinding of participants and researchers; 4) blinding of assessors of outcomes; 5) completeness of outcome data; 6) selective reporting bias; and 7) forms of other bias. Finally, the risk of bias was assessed as “high”, “low”, or “unclear” according to the above seven elements.

### Statistical Analysis

Review Manager (RevMan), version 5.3 (Nordic Cochrane Center, Cochrane Collaboration) was used to perform data analysis. Mean differences (MD) were used for continuous outcomes, risk ratios (RR) were used for dichotomous outcomes, and 95% confidence intervals (CI) were calculated for both variables. Heterogeneity among the trials was assessed using the Chi-square test (*p* < 0.10, defined as indicating significant heterogeneity) or *I* ^*2*^ (>50%). If substantial heterogeneity existed, a random effect model was applied; otherwise, a fixed effect model was applied. A narrative summary of the findings is provided for outcomes that could not be pooled. Subgroup analysis was performed for the duration of follow-up. Sensitivity analysis was conducted to evaluate the robustness of the results by excluding individual studies for forest plots. Publication bias was assessed by asymmetry in a funnel plot for GI at 4 weeks.

## Results

### Search Results and Study Characteristics

**FIGURE 1:**
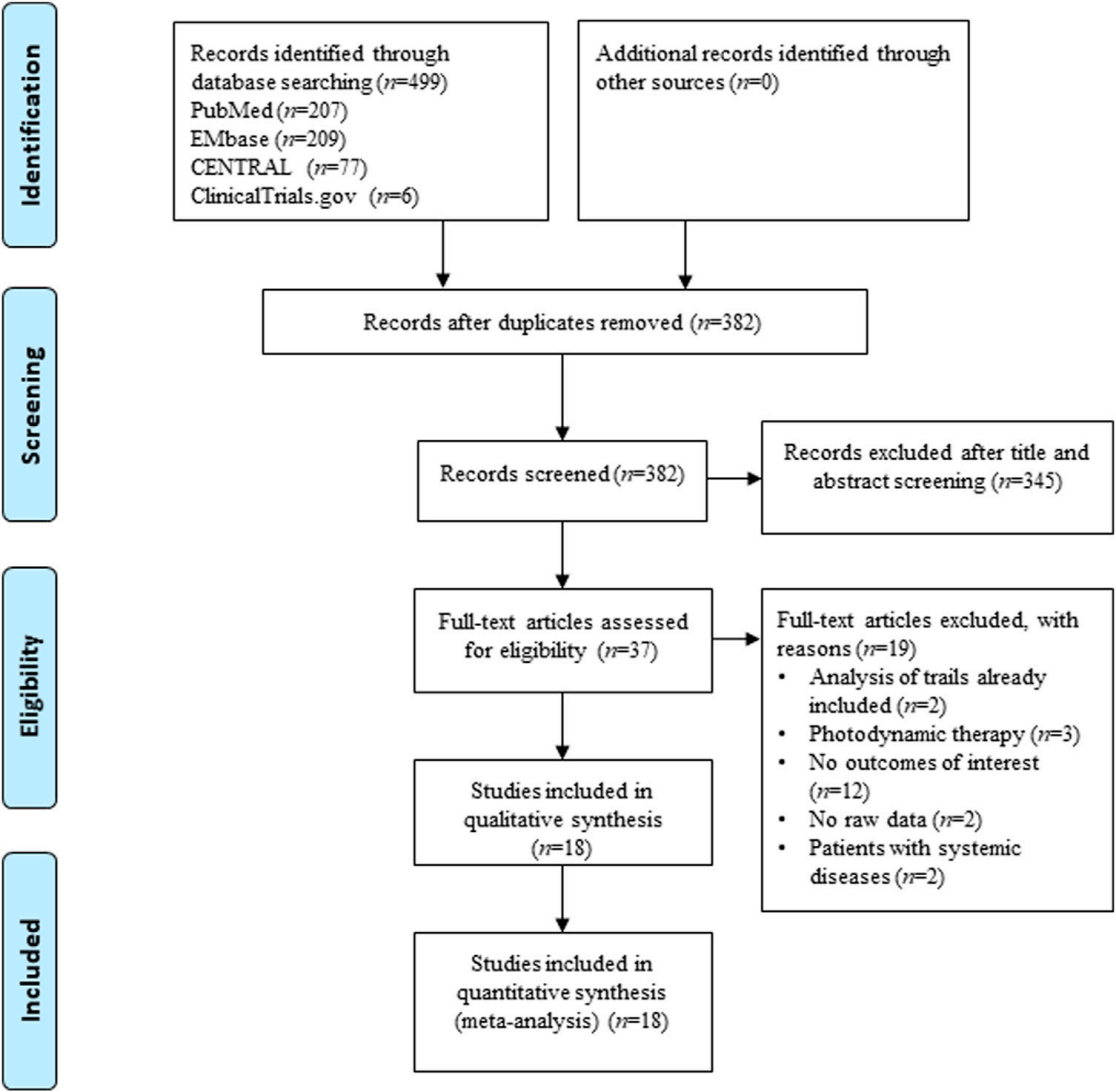
Flow diagram of literature screening.

Figure 1 shows the study selection process applied to identify the studies involved in this systematic review and meta-analysis. From the 499 potentially relevant reports identified, 37 studies proved potentially eligible after title and abstract screening. Following full text screening, 18 RCTs (Behal et al., 2011; Gottumukkala et al., 2013; Muglikar et al., 2013; Bhatia et al., 2014; Jaswal et al., 2014; Anuradha et al., 2015; Sreedhar et al., 2015; Arunachalam et al., 2017; Chatterjee et al., 2017; Singh et al., 2018; Jalaluddin et al., 2019; Kaur et al., 2019; Raghava et al., 2019; Guru et al., 2020; Mohammad, 2020; Pandey et al., 2021; Pérez-Pacheco et al., 2021; Rahalkar et al., 2021) involving 846 patients/sites were included in this meta-analysis. Table 1 displays the main characteristics of the 18 included studies. Seventeen studies were from India, and one was from Brazil. The demographic characteristics of the patients varied among the trials. However, the groups of each clinical trial were generally balanced with respect to demographic and clinical characteristics.

**TABLE 1:** Basic characteristics of included studies.

NO.	Study	Country	Study design	Age (years)	Male (%)	Sample size (treatment/control)	Diagnostic criteria	Study groups	Follow up (weeks)	Outcome indicators	Periodontal outcome evaluated
1	Gottumukkala et al. (2013)	India	SM	30–55	46.2%	23/23	Chronic Periodontitis: At least 3 sites with PPD ≥ 5 mm in three different quadrants and radiographic evidence of horizontal bone loss	1.NPT+1% curcumin subgingival irrigation	4, 12, and 24 weeks	BOP, PI, MI	The 1% curcumin showed a mild to moderate beneficiary effect to NPT
								2.NPT + saline			
2	Guru et al. (2020)	India	RCT	21–59	80.0%	15/15	Chronic Periodontitis: PPD of 5–7 mm with two or more teeth	1.NPT+2% curcumin nanogel	3 and 6 weeks	GI, PI, MI	The 2% curcumin gel showed an effective improvement of NPT in clinical parameters
								2.NPT			
3	Jalaluddin et al. (2019)	India	RCT	25–45	NA	20/20	Chronic Periodontitis: PPD ≥ 5 mm in different quadrants of the mouth	1.NPP+1% curcumin irrigation	4 and 8 weeks	GI, PI, MI	The curcumin combined with NPT show similar clinical parameters compared with NPT alone
								2.NPT			
4	Jaswal et al. (2014)	India	SM	21–55	80.0%	15/15	Chronic Periodontitis	1.NPT+2% gel	4 and 6 weeks	GI, PI	Th curcumin gel help in reduction of PPD.
								2.NPT			
5	Singh et al. (2018)	India	SM	30–50	55.0%	40/40	Chronic Periodontitis: < 30% of the sites assessed in the mouth demonstrate	1.NPT+5% curcumin chip	4 and 12 weeks	GI, PI, AE	Curcumin as an adjunct to NPT proved to be effective in the treatment of periodontitis
							attachment loss and bone loss; the	2.NPT			
							test sites with PPD 5–8 mm				
6	Behal et al. (2011)	India	SM	NA	NA	30/30	Chronic periodontitis with PD of 5–7 mm in at least 2 nonadjacent sites in different quadrants of the mouth	1.NPT+2% curcumin gel	4 and 6 weeks	GI, SBI, PI, MI, AE	2% curcumin gel can be effectively used as an adjunct to NPT and is more effective than NPT alone
								2.NPT			
7	Muglikar et al. (2013)	India	RCT	20–40	NA	10/10	Chronic generalized gingivitis manifesting change in the color and bleeding on probing but no signs of periodontitis	1.NPT+20% curcumin mouthwash	1, 2, and 3 weeks	GI, PI	20% curcumin mouthwash have statistically significantly better results compared with NPT alone
								2.NPT			
8	Bhatia et al. (2014)	India	SM	21–45	60.0%	25/25	Chronic periodontitis	1.NPT+1% curcumin gel	4, 12, 24 weeks	SBI, PI	1% curcumin gel provide significant improvements in clinical parameters when used as an adjunct to NPT compared with NPT alone
								2.NPT			
9	Anuradha et al. (2015)	India	SM	25–60	NA	30/30	Chronic periodontitis with pocket depth of 5–7 mm involving various quadrants of the mouth	1.NPT +10 mg/g curcumin gel	4 and 6 weeks	GI, PI	The curcumin gel as an adjunct to NPT with more effect achieved as NPT in periodontitis therapy
								2.NPT			
10	Chatterjee et al. (2017)	India	RCT	20–30	NA	50/50	Mild to moderate gingivitis with GI less than ≥1, PI ≥ 1	1.NPT+0.1% mouthwash	1, 2, 4 weeks	GI, SBI, PI, AE	0.1% mouthwash combined with NPT reveals statistically significant with NPT alone
								2.NPT			
11	Sreedhar et al. (2015)	India	SM	35–55	46.7%	15/15	Chronic periodontitis with at least one tooth with PPD > 5 mm in each quadrant	1.NPT+10 mg/g curcumin gel	4 and 12 weeks	SBI, PI, MI	The reduction in SBI scores was reflected in curcumin with NPT compared with NPT alone
								2.NPT			
12	Arunachalam et al. (2017)	India	RCT	25–60	40.0%	10/10	Chronic gingivitis	1.NPT + curcumin (0.1 curcumin mouthwash)	4w	GI, PI, BM	Curcumin mouthwash show significant reduction in PI and GI compared with NPT alone
								2.NPT			
13	Kaur et al. (2019)	India	RCT	20–60	NA	15/15	Moderate to severe chronic generalized periodontitis having at least four sites with pocket probing depth of 5 mm or more in one or both of arches	1.NPT+1% curcumin gel	4 and 12 weeks	SBI, PI, BM, AE	Single application of curcumin gel has limited added benefit over NPT in treatment of chronic periodontitis
								2.NPT			
14	Raghava et al. (2019)	India	SM	25–40	50.0%	10/10	Chronic periodontitis having a PPD ≥ 5 mm	1.NPT + curcumin gel	4 weeks	GI, PI	The local application of curcumin gel when used in conjunction with NPT showed a significant improvement in PI and PPD compared with NPT alone
								2.NPT			
15	Mohammad (2020)	India	RCT	36.73 ± 6.22	36.7%	30/30	Chronic periodontitis: PPD ≥ 4 mm and CAL ≥ 2 mm in at least 40% of the analyzed sites	1.NPT+10 mg/g curcumin gel	4 weeks	GI, BOP, PI, BM	Curcumin gel resulted in a more significant reduction in clinical parameters compared to NPT alone
								2.NPT			
16	Pérez-Pacheco et al. (2021)	Brazil	SM	37–62	70.0%	40/40	Generalized periodontitis with stage III and Grade A, presenting two non-adjacent sites with PPD ≥ 5 mm and BOP in two different quadrants, and bone loss confirmed by radiographs	1.NPT+0.05 mg/ml curcumin gel	4, 12, 24 weeks	BOP, MI, BM, AE	Local administration of curcumin had no additional benefits to NPT.
								2.NPT			
17	Pandey et al. (2021)	India	RCT	20–60	NA	30/30	Gingivitis	1.NPT + curcumin gel	1, 2, and 3 weeks	GI, SBI, PI	Curcumin gel has significant effect in the treatment of gingivitis as an adjunct to NPT
								2.NPT			
18	Rahalkar et al. (2021)	India	SM	37–57	33.3%	15/15	PPD ≥ 5 mm and ≤ 8 mm at three nonadjacent sites in different quadrants of the mouth	1.NPT + curcumin gel	4 weeks	GI, SBI, PI, BM	Curcumin showed significant reduction in PI in curcumin group when compared with NPT
								2.NPT			

RCT, randomized clinical trial; SM, split-mouth design; PPD, periodontal probing depth; CAL, clinical attachment level; SRP, scaling and root planning; GI, gingival index; SBI, sulcus bleeding index; PI, plaque index; BOP, bleeding on probing; NA, not available; BM, biochemical marker; MI, microbiologic indicator; AE, adverse effect.

### Quality Assessment of the Included Studies

**FIGURE 2:**
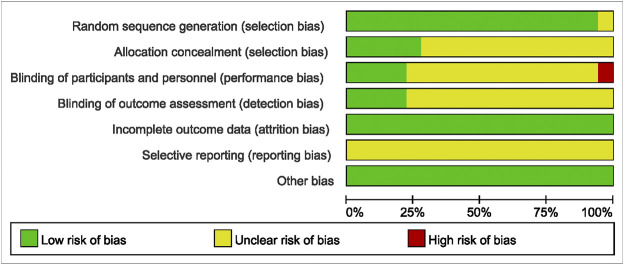
Risk of bias graph.

**FIGURE 3:**
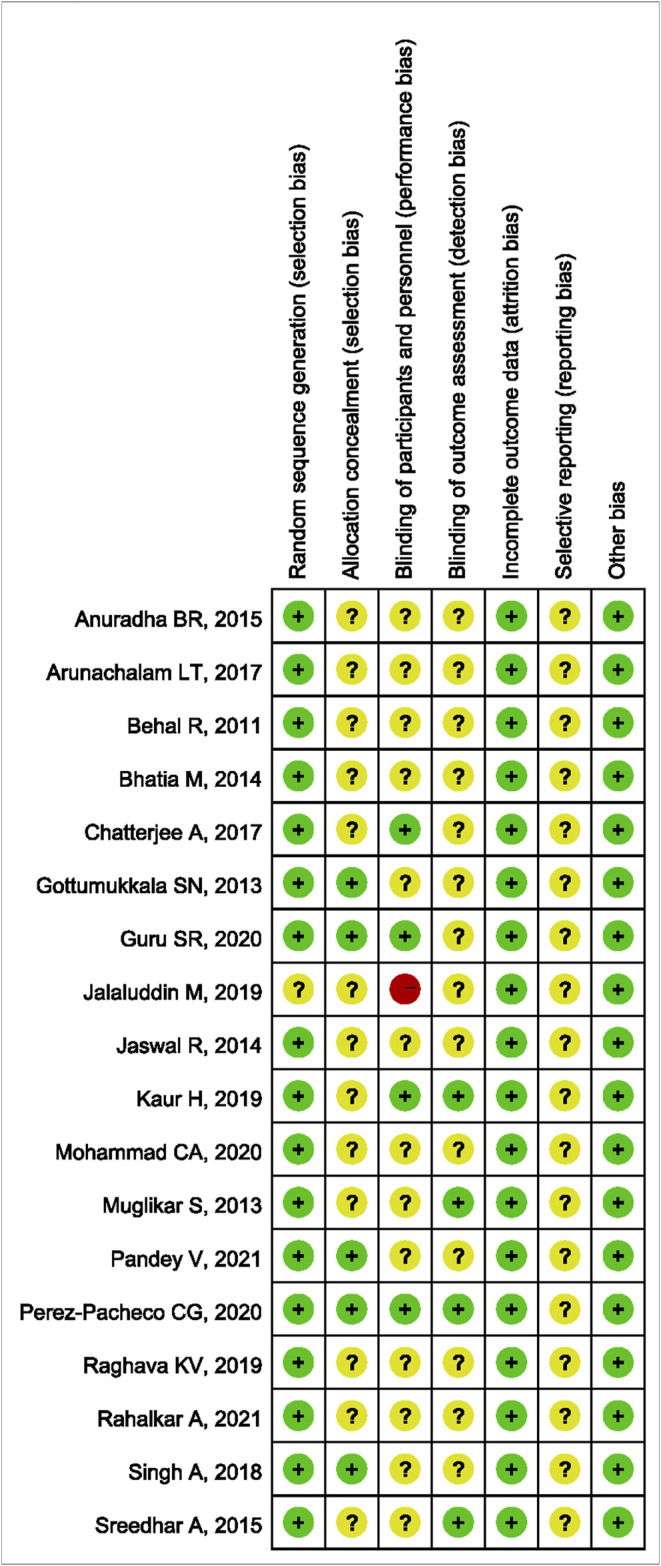
Risk of bias summary.

The methodological quality results are shown in Figure 2 and Figure 3. Only one (Jalaluddin et al., 2019) study did not mention randomized allocation. Five studies (Gottumukkala et al., 2013; Singh et al., 2018; Guru et al., 2020; Pandey et al., 2021; Pérez-Pacheco et al., 2021) mentioned allocation concealment. Participants and trial staff were not blinded in one study (Jalaluddin et al., 2019), and 13 other studies (Behal et al., 2011; Gottumukkala et al., 2013; Muglikar et al., 2013; Bhatia et al., 2014; Jaswal et al., 2014; Anuradha et al., 2015; Sreedhar et al., 2015; Arunachalam et al., 2017; Singh et al., 2018; Raghava et al., 2019; Mohammad, 2020; Pandey et al., 2021; Rahalkar et al., 2021) failed to mention blinding of participants and personnel. Only four studies (Muglikar et al., 2013; Sreedhar et al., 2015; Kaur et al., 2019; Pérez-Pacheco et al., 2021) that reported outcome assessors were blinded. All studies had complete data and consistent outcomes, as described in the methods section. No study had described the registration of RCTs.

### Primary Outcomes

#### GI

**FIGURE 4:**
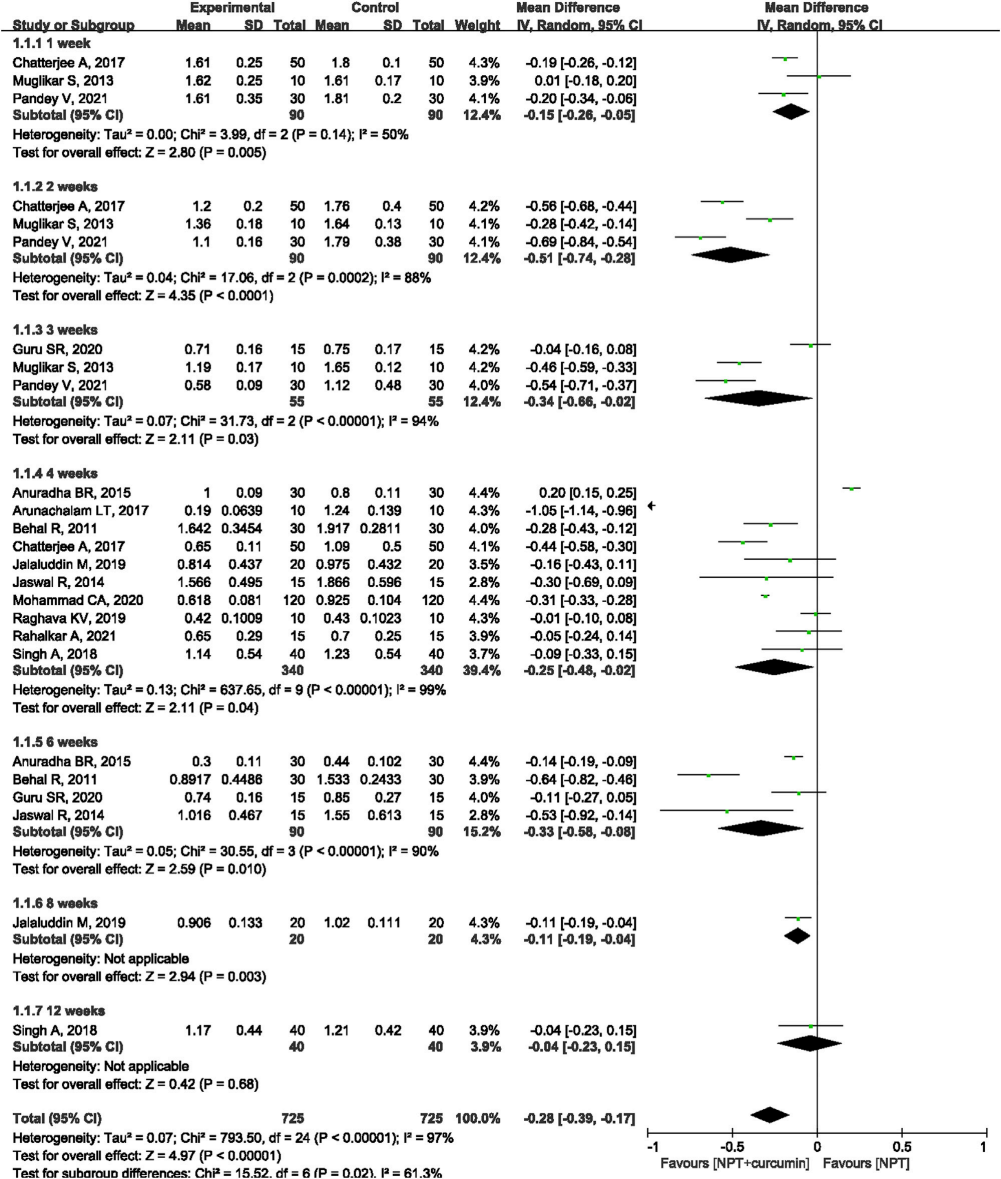
Forest plot of the effects of curcumin + NPT versus NPT on GI.

Thirteen RCTs (Behal et al., 2011; Muglikar et al., 2013; Jaswal et al., 2014; Anuradha et al., 2015; Arunachalam et al., 2017; Chatterjee et al., 2017; Singh et al., 2018; Jalaluddin et al., 2019; Raghava et al., 2019; Guru et al., 2020; Mohammad, 2020; Pandey et al., 2021; Rahalkar et al., 2021) reported the GI outcome. Meta-analysis with the random-effects model revealed that there were statistically significant differences in reducing GI between curcumin as an adjunct and NPT alone at 1 week (MD = −0.15, 95%CI −0.26 to −0.05, *p* = 0.005), 2 weeks (MD = −0.51, 95%CI −0.74 to −0.28, *p* < 0.000 1), 3 weeks (MD = −0.34, 95%CI −0.66 to −0.02, *p* = 0.03), 4 weeks (MD = −0.25, 95%CI −0.48 to −0.02, *p* = 0.04) and 6 weeks (MD = −0.33, 95%CI -0.58 to −0.08, *p* = 0.01) (Figure 4). Only one study (Jalaluddin et al., 2019) reported that curcumin as an adjunct had a significantly higher reduction in GI (MD = −0.11, 95%CI −0.19 to −0.04, *p* = 0.003) than NPT alone at the 8-week evaluation. Another study (Singh et al., 2018) showed that there was no significant difference between curcumin as an adjunct to NPT and NPT alone at 12 weeks (MD = −0.04, 95%CI −0.23 to −0.15, *p* = 0.68).

#### SBI

**FIGURE 5:**
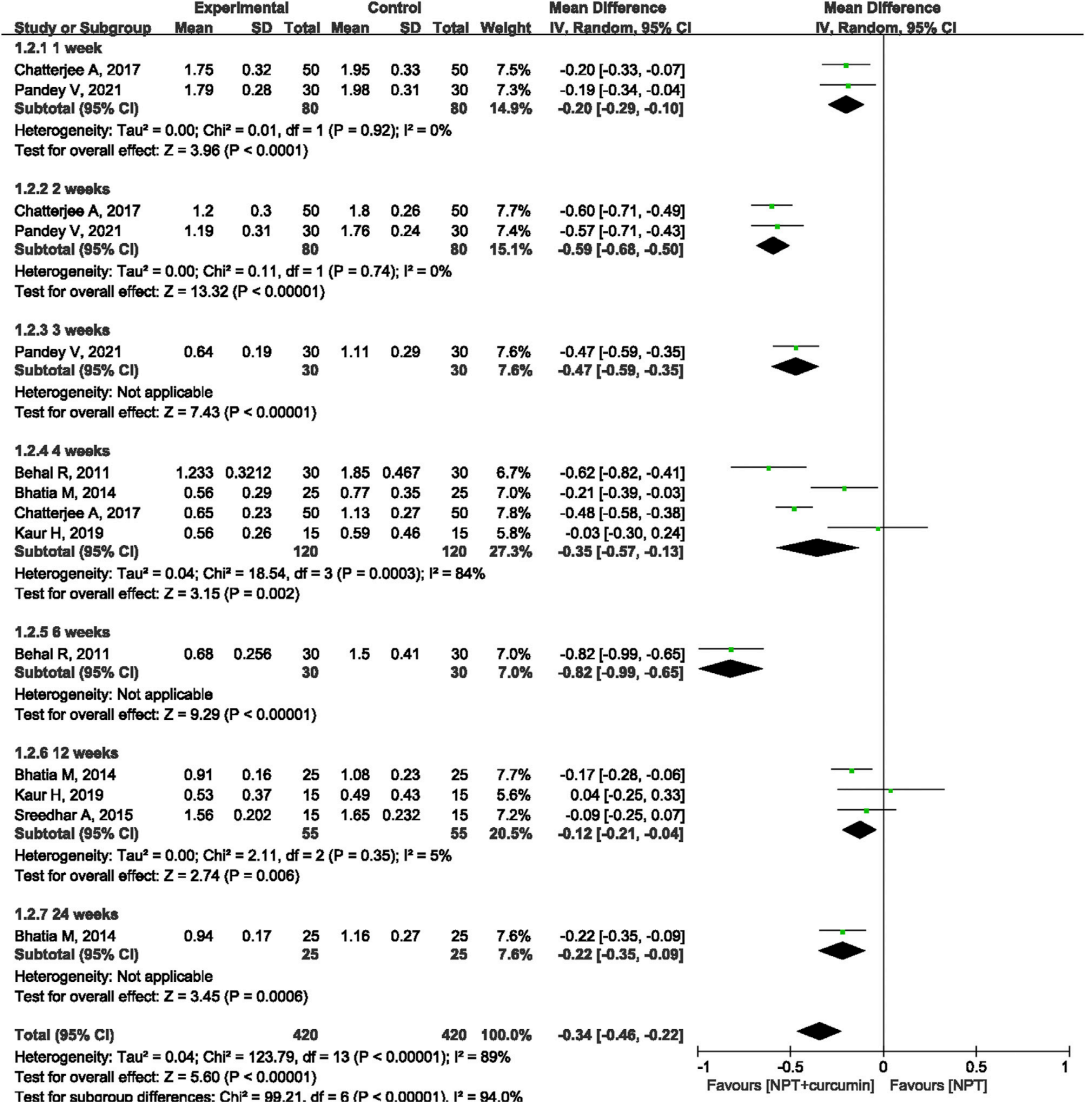
Forest plot of the effects of curcumin + NPT versus NPT on SBI.

Seven RCTs (Behal et al., 2011; Bhatia et al., 2014; Sreedhar et al., 2015; Chatterjee et al., 2017; Kaur et al., 2019; Pandey et al., 2021; Rahalkar et al., 2021) involving 360 patients/sites reported the SBI index. Meta-analysis with the random-effects model revealed that there were statistically significant differences in reducing SBI between curcumin as an adjunct and NPT alone at 1 week (MD = −0.20, 95%CI −0.29 to −0.10, *p* < 0.0001), 2 weeks (MD = −0.59, 95%CI −0.68 to −0.50, *p* < 0.00001), 4 weeks (MD = −0.35, 95%CI −0.57 to −0.13, *p* = 0.002) and 12 weeks (MD = −0.12, 95%CI −0.21 to −0.04, *p* = 0.006) (Figure 5). There were also statistical differences between curcumin as an adjunct to NPT and NPT alone at 6 weeks (MD = -0.82, 95%CI -0.99 to -0.65, *p* < 0.0001) and 24 weeks (MD = -0.22, 95%CI -0.35 to -0.09, *p* = 0.0006), but there was only one study for each follow-up time.

#### BOP

**FIGURE 6:**
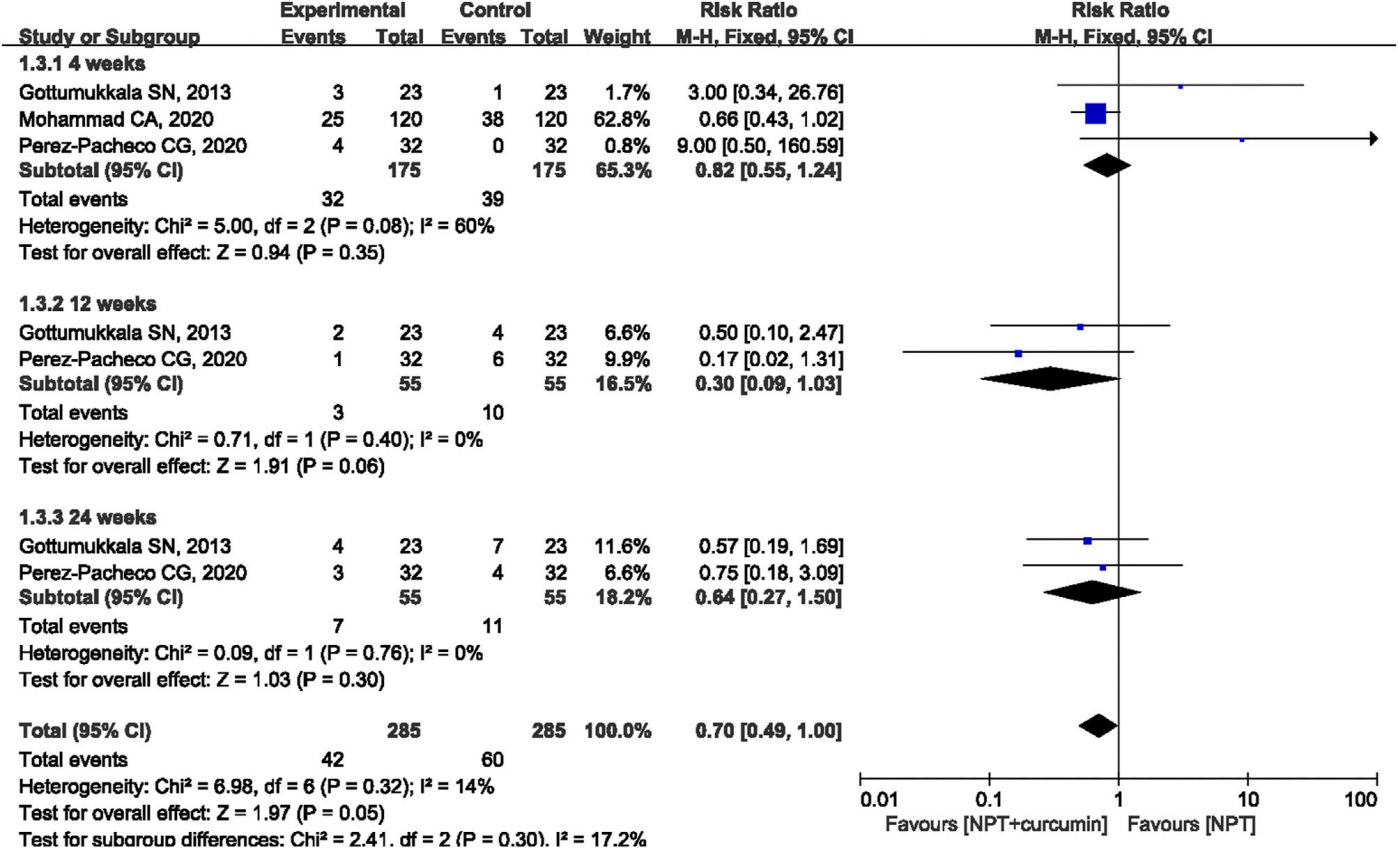
Forest plot of the effects of curcumin + NPT versus NPT on BOP.

Three RCTs (Gottumukkala et al., 2013; Mohammad, 2020; Pérez-Pacheco et al., 2021) reported the BOP outcome. The results of the meta-analysis revealed that there were no statistically significant differences in reducing BOP between curcumin as an adjunct and NPT alone at 4 weeks (MD = 0.82, 95%CI 0.55 to 1.24, *p* = 0.35), 12 weeks (MD = −0.30, 95%CI 0.09 to 1.03, *p* = 0.06), and 24 weeks (MD = 0.64, 95%CI 0.27 to 1.50, *p* = 0.30) (Figure 6).

### Secondary Outcomes

#### PI

**FIGURE 7:**
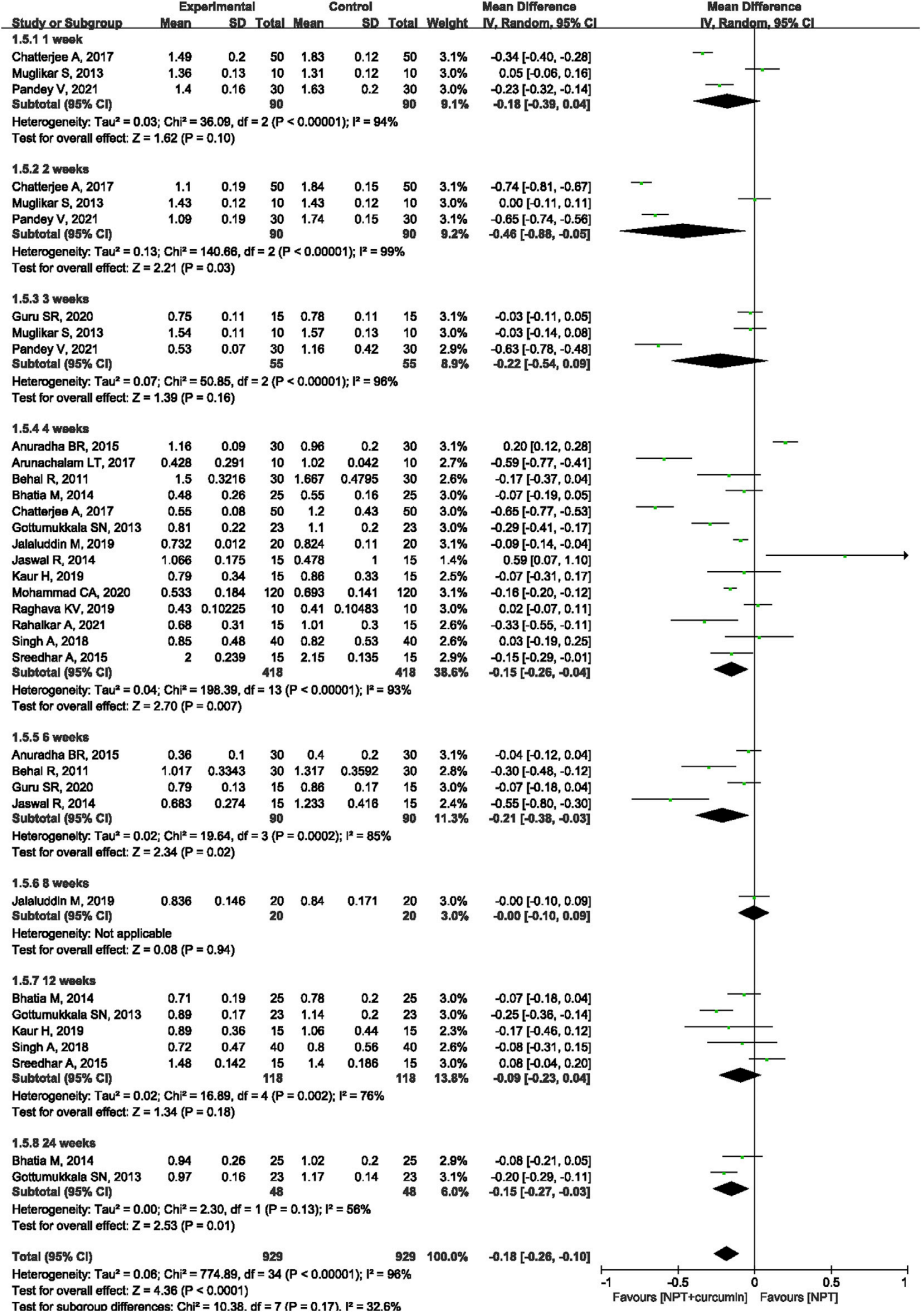
Forest plot of the effects of curcumin + NPT versus NPT on PI.

Seventeen RCTs (Behal et al., 2011; Gottumukkala et al., 2013; Muglikar et al., 2013; Bhatia et al., 2014; Jaswal et al., 2014; Anuradha et al., 2015; Sreedhar et al., 2015; Arunachalam et al., 2017; Chatterjee et al., 2017; Singh et al., 2018; Jalaluddin et al., 2019; Kaur et al., 2019; Raghava et al., 2019; Guru et al., 2020; Mohammad, 2020; Pandey et al., 2021; Rahalkar et al., 2021) reported the PI outcome. Meta-analysis with the random-effects model showed that there were statistically significant differences in reducing PI between NPT and curcumin an adjunct to NPT at 2 weeks (MD = −0.46, 95%CI −0.88 to −0.05, *p* = 0.03), 4 weeks (MD = −0.15, 95%CI −0.26 to −0.04, *p* = 0.007), 6 weeks (MD = −0.21, 95%CI −0.38 to −0.03, *p* = 0.02) and 24 weeks (MD = −0.15, 95%CI -0.27 to −0.03, *p* = 0.01). However, there were no significant differences at 1 week (MD = −0.18, 95%CI −0.39 to 0.04, *p* = 0.10), 3 weeks (MD = −0.22, 95%CI −0.54 to 0.09, *p* = 0.16), and 12 weeks (MD = −0.09, 95%CI −0.23 to 0.04, *p* = 0.18) (Figure 7).

#### Microbiological Indicators

Seven of the recruited studies compared subgingival microbial levels between NPT and NPT with curcumin (Behal et al., 2011; Gottumukkala et al., 2013; Bhatia et al., 2014; Sreedhar et al., 2015; Guru et al., 2020; Pérez-Pacheco et al., 2021; Rahalkar et al., 2021). Significant reductions in bacterial loads, such as *Porphyromonas gingivalis (P. gingivalis), Treponema denticola (T. denticola), Tannerella forsythia (T. forsythia), Prevetella intermedia (P. intermedia), Fusobacterium nucleatum (F. nucleatum), Actinobacillus actinomycetemcomitans (A. actinomycetemcomitans)* and some other periodontal pathogens (Behal et al., 2011; Gottumukkala et al., 2013; Bhatia et al., 2014; Sreedhar et al., 2015; Guru et al., 2020; Pérez-Pacheco et al., 2021; Rahalkar et al., 2021), were shown once curcumin was used as an adjuvant to NPT, whereas another study reported no benefit in comparison with NPT alone (Rahalkar et al., 2021).

#### Inflammatory Factors

Data from three articles were demonstrated (Kaur et al., 2019; Mohammad, 2020; Pérez-Pacheco et al., 2021). Clinical studies on NPT combined with curcumin report mixed results: one of the studies indicated that there was no difference in GCF cytokine levels, such as IL-1 and TNF-α, but other studies reported no benefit in comparison with NPT alone (Kaur et al., 2019; Pérez-Pacheco et al., 2021).

#### Safety

No adverse events were reported during the follow-up in the included studies (Behal et al., 2011; Chatterjee et al., 2017; Singh et al., 2018; Kaur et al., 2019; Pérez-Pacheco et al., 2021). Other studies did not mention adverse events. However, a portion of individuals reported curcumin mouthwash has an unacceptable taste (Chatterjee et al., 2017).

### Sensitivity Analysis

All results showed good consistency in both the fixed-effects and random-effects models. The overall effect direction did not change after deleting one study each time for GI and SBI. Sensitivity analysis results indicated that the outcomes were not reversed by removing any study, which had relatively good stability.

### Publication Bias

**FIGURE 8:**
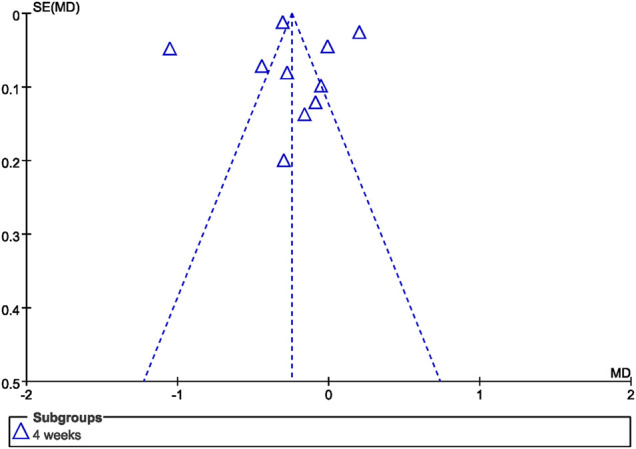
Funnel plot of GI at 4 weeks.

The funnel plot of GI at 4 weeks demonstrated no significant asymmetrical distribution (Figure 8).

## Discussion

In recent years, curcumin has been used as an adjunct to non-surgical periodontal treatment. However, the efficacy of curcumin in periodontal therapy remains controversial. This study aimed to evaluate the anti-inflammatory efficacy of curcumin as an adjunct to non-surgical periodontal treatment (NPT) by means of a systematic review. The results of the meta-analysis revealed that there were statistically significant differences in reducing GI between NPT alone and curcumin as an adjunct to NPT at the 1-, 2-, 3-, four- or 6-week follow-ups. Significant differences were also found in reducing SBI between these two groups at weeks 1, 2, 4 and 12. However, there were no statistically significant differences in reducing BOP between curcumin as an adjunct and NPT alone at 4, 12, and 24 weeks. Thus, curcumin has a similar effect on reducing GI and SBI compared with NPT alone when applied as an adjunct to NPT for treating periodontal disease.

In the present study, GI, SBI and BOP were used as clinical indications of periodontal inflammation. GI is based on a combination of visual evaluation and mechanical stimulus of marginal periodontal tissues. GI scores demonstrated a significant correlation with histological parameters of inflammation during the development of periodontal disease (da Silva et al., 2021). SBI provides an objective assessment for detecting early inflammatory changes in inflammatory lesions, which are sometimes difficult to visually examine (Newman, 2015). Therefore, the GI and SBI appear to be the most useful and the most easily transferred to clinical practice (Newman, 2015). This systematic review showed that NPT + curcumin could significantly reduce the GI and SBI at the 1-, 2-, 3-, 4-, and 6-week follow-ups compared to the group receiving only mechanical debridement as the treatment modality, demonstrating that using adjunctive curcumin showed better improvement in the reduction of gingival inflammation and bleeding. However, this study revealed that there were no statistically significant differences in reducing BOP between these two groups. The result of SBI varies from that of BOP because color changes may be present without BOP (Greenstein, 1984). Meanwhile, the limited sample sizes may be another factor.

The mechanism of periodontal disease involves the production of several inflammatory mediators. Periodontal pathogens activate NF-κB, Janus kinase (JAK)/signal transducer, activator of transcription (STAT), mitogen-activated protein kinases (MAPK), and other signaling pathways and produce inflammatory cytokines such as IL-6, TNF-α and IL-1β to promote inflammation (Li et al., 2021). Curcumin, the active ingredient in turmeric, has various anti-inflammatory properties and may delay the disease process of periodontal disease in its initial stages. It has been shown to suppress the NF-κB pathway in human gingival fibroblasts in early stages and thus may inhibit *P. gingivalis* LPS-induced COX-2 synthesis (Hu et al., 2013) and the production of TNF-α, IL-8 and IL-6 by inhibiting NF-κB activation in mast cells (Kong et al., 2018). Additionally, curcumin could exert an anti-inflammatory effect by directly inhibiting the JAK/STAT signaling pathway and phosphorylation of p38 MAPK, thereby reducing the expression of iNOS, COX-2, monocyte chemoattractant protein-1 (MCP-1), and intercellular adhesionmolecule-1(ICAM-1) (Guimarães et al., 2013; Boyle et al., 2015) to reduce the inflammatory response.

Previous studies have indicated that curcumin may have an additional antimicrobial effect, although the summaries of the included articles are inconclusive. Dental plaque is an important factor in the pathological process of periodontal disease. *In vitro* studies have proven that curcumin can inhibit the growth of periodontal pathogens (such as *A. actinomycetemcomitans, F. nucleatum, and P. gingivalis*) under planktonic and biofilm conditions (Shahzad et al., 2015). The decrease in periodontal pathogens and LPS in Gram-negative bacterial walls could inhibit innate and adaptive immune responses in periodontal tissues. This effect could also explain why curcumin could suppress the inflammatory process in periodontal tissue.

Curcumin is a polyphenol found in the rhizome of turmeric, which is a spice commonly used in Asian cooking. The utilization of curcumin has proven to be safe for both animals and humans, even at high doses (Shankar et al., 1980; Lao et al., 2006). Therefore, no adverse events were reported during the follow-up in the included studies (Chatterjee et al., 2017; Singh et al., 2018; Kaur et al., 2019; Pérez-Pacheco et al., 2021).

This review has several limitations. First, this study had high statistical heterogeneity, which could not be explained by the duration of follow-up. This seems to be the consequence of both methodological and clinical heterogeneity. The heterogeneity resulted from factors such as variation in disease severity, delivery method and different concentrations of the treatments used. Unfortunately, the included articles did not provide sufficient details for us to analyze the influences of these factors. Second, only the PubMed, Embase, CENTRAL and ClinicalTrials.gov databases were searched in our meta-analysis, which could leave out some literature that may influence the final results. Third, non-English articles were excluded because we cannot understand other languages very accurately. Fourth, almost all included studies are from India. Although studies on curcumin have been conducted in many countries, clinical studies aiming to evaluate the efficacy of curcumin were mainly conducted in India. Multi-center clinical trials will definitely help to verify the clinical application of curcumin. Finally, given the small sample size and limited number of studies on certain outcomes, the results might be insufficient to ensure a significant difference.

Based on the limitations above, more high-quality, registered RCTs with a large-scale sample are needed. In addition, clinical trials regarding the use of curcumin should standardize the severity of periodontal disease and treatment methods to explore the clinical effectiveness of curcumin. Safety evaluations of curcumin also need more attention.

## Conclusion

In conclusion, based on the current evidence, the results of this systematic review and meta-analysis show that curcumin demonstrates anti-inflammatory efficacies in terms of reducing GI and SBI compared with NPT alone. Moreover, curcumin is a natural herbal medicine with few side effects, and it is a good candidate as an adjunct treatment for periodontal disease. Limited by the quantity and quality of the included studies, further high-quality studies with a large-scale sample are needed to confirm the anti-inflammatory efficacy and safety of curcumin as an adjunct to NPT.

## Data Availability

The original contributions presented in the study are included in the article/Supplementary Material, further inquiries can be directed to the corresponding author.
