# Supplementary material for: Anti-Inflammatory Efficacy of Curcumin as an Adjunct to Non-Surgical Periodontal Treatment: A Systematic Review and Meta-Analysis
Source: Front Pharmacol. 2022 Jan 24;13:808460. doi: 10.3389/fphar.2022.808460 (PMC8819153; doi:10.3389/fphar.2022.808460)
Supplement: Supplementary file 1 [file DataSheet1.PDF]

Supplementary Material

1 Search Strategy for Embase

EMbase-Ovid（2021.7.21）
Embase <1974 to 2021 July 20>
1 exp periodontitis/ 45443
2 Periodontitis.ti. or Periodontitis.ab. or Periodontitis.kw. 31276
3 Periodontal.ti. or Periodontal.ab. or Periodontal.kw. 62591
4 Parodontosis.ti. or Parodontosis.ab. or Parodontosis.kw. 173
5 Parodontoses.ti. or Parodontoses.ab. or Parodontoses.kw. 2
6 gingival disease.ti. or gingival disease.ab. or gingival disease.kw. 187
7 gum inflammation.ti. or gum inflammation.ab. or gum inflammation.kw. 43
8 Periodontitides.ti. or Periodontitides.ab. or Periodontitides.kw. 1
9 Pericementitis.ti. or Pericementitis.ab. or Pericementitis.kw. 3
10 Gingivosis.ti. or Gingivosis.ab. or Gingivosis.kw. 1
11 Gingivoses.ti. or Gingivoses.ab. or Gingivoses.kw. 0
12 gingivitis.ti. or gingivitis.ab. or gingivitis.kw. 8541
13 1 or 2 or 3 or 4 or 5 or 6 or 7 or 8 or 9 or 10 or 11 or 12 90195
14 exp curcumin/ 28173
15 curcumin.ti. or curcumin.ab. or curcumin.kw. 21373
16 curcuma.ti. or curcuma.ab. or curcuma.kw. 5478
17 Turmeric.ti. or Turmeric.ab. or Turmeric.kw. 4827
18 Diferuloylmethane.ti. or Diferuloylmethane.ab. or Diferuloylmethane.kw. 486
19 Demethoxycurcumin.ti. or Demethoxycurcumin.ab. or Demethoxycurcumin.kw. 469
20 Bisdemethoxycurcumin.ti. or Bisdemethoxycurcumin.ab. or Bisdemethoxycurcumin.kw. 440
21 14 or 15 or 16 or 17 or 18 or 19 or 2033571
22 13 and 21 209

2 Search Strategy for Cochrane Library

Cochrane Library-Ovid （2021.7.21）
EBM Reviews - Cochrane Central Register of Controlled Trials <June 2021>
EBM Reviews - Cochrane Database of Systematic Reviews <2005 to July 14, 2021>
1 exp Periodontitis/ 3033
2 Periodontal.ti. or Periodontal.ab. or Periodontal.kw. 8184
3 Periodontitis.ti. or Periodontitis.ab. or Periodontitis.kw. 4805
4 Parodontosis.ti. or Parodontosis.ab. or Parodontosis.kw. 0
5 Parodontoses.ti. or Parodontoses.ab. or Parodontoses.kw. 0
6 gingival disease.ti. or gingival disease.ab. or gingival disease.kw. 32
7 gum inflammation.ti. or gum inflammation.ab. or gum inflammation.kw. 16
8 Periodontitides.ti. or Periodontitides.ab. or Periodontitides.kw. 0
9 Pericementitis.ti. or Pericementitis.ab. or Pericementitis.kw. 0
10 Gingivosis.ti. or Gingivosis.ab. or Gingivosis.kw. 0
11 Gingivoses.ti. or Gingivoses.ab. or Gingivoses.kw. 0

12 gingivitis.ti. or gingivitis.ab. or gingivitis.kw. 2617
13 1 or 2 or 3 or 4 or 5 or 6 or 7 or 8 or 9 or 10 or 11 or 12 12094
14 exp curcumin/ 431
15 curcumin.ti. or curcumin.ab. or curcumin.kw. 1244
16 curcuma.ti. or curcuma.ab. or curcuma.kw. 354
17 Turmeric.ti. or Turmeric.ab. or Turmeric.kw. 491
18 Diferuloylmethane.ti. or Diferuloylmethane.ab. or Diferuloylmethane.kw. 9
19 Demethoxycurcumin.ti. or Demethoxycurcumin.ab. or Demethoxycurcumin.kw. 23
20 Bisdemethoxycurcumin.ti. or Bisdemethoxycurcumin.ab. or Bisdemethoxycurcumin.kw. 20
21 14 or 15 or 16 or 17 or 18 or 19 or 201744
22 13 and 21 77

3 Search Strategy for ClinicalTrials.gov

ClinicalTrials.gov （2021.7.21）
Condition or disease: (periodontal) OR (Parodontosis) OR (Parodontoses) OR (gingival disease) OR (Gingivosis) OR (Gingivoses) OR (gingivitis) OR (gum inflammation) OR (periodontitis) OR (Periodontitides) OR (Pericementitis)
Other terms: (curcumin) OR (curcuma) OR (Turmeric) OR (Diferuloylmethane) OR (Demethoxycurcumin) OR (Bisdemethoxycurcumin) OR (CMC 2.24)

4 Search Strategy for PubMed

PubMed （2021.7.21）
((((((((curcumin[MeSH Terms]) OR (curcumin)) OR (curcuma)) OR (Turmeric)) OR (Diferuloylmethane)) OR (Demethoxycurcumin)) OR (Bisdemethoxycurcumin)) OR (CMC 2.24)) AND ((((((((((((Periodontitis[MeSH Terms]) OR (Periodontitis)) OR (Periodontal)) OR (Parodontosis)) OR (Parodontoses)) OR (gingival disease)) OR (gingival disease)) OR (Periodontitides)) OR (Periodontitides)) OR (Gingivosis)) OR (Gingivosis)) OR (gingivitis))

5 Supplementary Table S1: Supplement Details respecting to the information of curcumin

Study	Formulation	Species, source, concentration	Form	Quality control reported? (Y/N)	Chemical analysis reported? (Y/N)
Gottumukkala SN, 2013	Self-made formula	1mg curcumin extract dissolved in 5ml ethanol; 95ml glycerol	solution	N	N
Guru SR, 2020	Self-made formula	2% curcumin powder (Konark Herbals and Health Care, Mumbai, Maharashtra, India); 20% pluronic nanogel	gel	N	N

Jalaluddin M, 2019	Self-made formula	1000 μg curcumin in 5ml ethanol and 95ml glycerol	solution	N	N
Jaswal R, 2014	Self-made formula	2% turmeric extract, 20% pluronic polymer, water	gel	N	N
Singh A, 2018	Self-made formula	5% curcumin in 1000mg hydroxy propyl cellulose matrix	chip	N	N
Behal R, 2011	Self-made formula	2% turmeric extract; 20% pluronic polymer; water	gel	N	N
Muglikar S, 2013	Self-made formula	5.139g curcumin extract, 50ml water	solution	N	N
Bhatia M, 2014	Self-made formula	100mg curcumin (Natural Remedies Pvt Limited), 80ml distilled water; 22gm pluronic F-127 (Sigma-Aldrich USA)	gel	The curcumin gel was dissolved in methanol, and its absorbance was measured spectrophotometrically at 425nm	N
Chatterjee A, 2017	Self-made formula	curcumin(diferuloylmethane) that comprises 0.3%-5.4% raw turmeric, tumerone, atlantone, and zingiberone	solution	The curcumin mouthwash was carried out by Sanso Syre pharmaceutical company	N
Arunachalam LT, 2017	Self-made formula	10mg curcumin extract, 100ml distrilled water, 0.005% flavoring agent (peppermint oil)	solution	N	N
Perez-Pacheco CG, 2020	Self-made formula	0.5 mg/mL curcumin (Sigma-Aldrich Co. cat#C1386, Lot#081M1611V) ethanol solution, polyglycolic and poly-lactic acids (PGLA/PLA)	nanoparticles	The curcumin loaded nanoparticles were prepared by Dr. Antonio Claudio Tedesco (Department of Chemistry, University of Sao Paulo)	N
Sreedhar A, 2015	curenext oral gel®	Abbott Healthcare Pvt., Ltd Mumbai, India	10mg of curcuma	The curcumin was prepared according to	N

Anuradha BR, 2015		longa extract/g of gel	Abbott Healthcare Pvt. Ltd pharmacopeia
Kaur, 2019			
Rahalkar A, 2021			
Raghava KV, 2019			
Mohammad CA, 2020			
